# Organisation and timeline of measures in French psychiatric establishments during the first wave of the COVID-19 epidemic: EvOlu’Psy study

**DOI:** 10.1186/s12888-021-03293-0

**Published:** 2021-06-02

**Authors:** Guillaume Legrand, Catherine Boisgard, Bernard Canac, Zuzana Cardinaud, Michela Giugiario Gorla, Elisabeth Gregoire, Caroline Jamon, Tarik Oussal, Pascal Vaury

**Affiliations:** 1 Association Hospitalière Sainte-Marie, Centre Hospitalier Sainte-Marie de Clermont-Ferrand, 33 Rue Gabriel Péri, 63000 Clermont-Ferrand, France; 2 Association Hospitalière Sainte-Marie, Centre Hospitalier Sainte-Marie de Rodez, Cayssiols, 12510 Rodez, France; 3 Association Hospitalière Sainte-Marie, Centre Hospitalier Sainte-Marie de Nice, 87 Avenue Joseph Raybaud, 06100 Nice, France; 4 Association Hospitalière Sainte-Marie, Centre Hospitalier Sainte-Marie du Puy-en-Velay, 50 Route de Montredon, 43009 Le Puy-en-Velay, France; 5 Association Hospitalière Sainte-Marie, Centre Hospitalier Sainte-Marie de Rodez, 19 Cours du Temple, 07000 Privas, France

**Keywords:** Mental health departments, COVID-19, Organisation, Audit, Pandemic, Response, Restriction

## Abstract

**Background:**

The onset of COVID-19 required rapid organisational changes in the mental health domain. Most mental health-care departments appear to have set up infection control measures and also organised planning, coordination and measures that enabled them to provide psychiatric care in a restrictive environment. Our objective was to assess the organisation by psychiatric facilities in France of their response to COVID-19, during the first wave.

**Methods:**

In June 2020, a cross-sectional study was performed by an audit with 48 items which was proposed to 331 hospitals in metropolitan France with a capacity for full-time, that is, inpatient psychiatric hospitalisation of adults.

**Results:**

Of the 331 establishments contacted, 94 (28.4%) agreed to respond to the survey questionnaire. Full-time inpatient hospitalisation was completely or partially maintained by 94.7% (*n* = 89) of facilities. Specific measures concerning respect for patients’ rights were reported by 58% (*n* = 55) of establishments. Overall, 74.5% (*n* = 70) had set up a dedicated channel of care for patients at risk of severe COVID-19, and 52.1% (*n* = 49) a system for routine screening at admission for these risk factors. Nearly half the establishments (48.9%, *n* = 46) reported they had set up specific training programmes for patients about barrier measures and social distancing.

**Conclusions:**

French psychiatric establishments on the whole were able to provide a necessary reorganisation of their management of patients and their families, regardless of facility status. Patients’ rights nonetheless seem to have not received the attention they merited during the early pandemic period. Somatic management of patients with mental illness must absolutely be improved.

**Supplementary Information:**

The online version contains supplementary material available at 10.1186/s12888-021-03293-0.

## Background

The onset of the pandemic of SARS-CoV2 (Coronavirus-19) and the illness it causes (COVID-19) required rapid organisational changes in the mental health domain [1]. Patients with psychiatric disorders generally present a higher risk of infection and specially to SARS-CoV2 than people without mental disorders and may be at greater risk of developing multiorgan failure and more likely to die in an intensive care unit (ICU) as they suffer from untreated comorbidities (diabetes, obesity, hypertension) [2, 3].

Among outpatients, the lockdown measures (home confinement), the remoteness of daily activities, and the imposition of social distancing have particularly affected those with psychiatric disorders, with an increase in anxiety and depression disorders and high rates of sleep disorders and post-traumatic stress disorders (PTSD) [4]. Moreover, based on an extensive literature review, Chevance et al., demonstrated that early hospital discharges and breaks in in-person psychiatric follow-up were frequent, with consequences including relapse, suicidal behaviour, difficulties in access to care, and social isolation [5].

In inpatient care, compliance with social distancing and other barrier measures have appeared to be challenges in psychiatry departments: patients receiving care in these departments may have cognitive and behavioural vulnerabilities and learning difficulties, all of which can impede the application of these measures [6]. Moreover, although scientific data on the topic are sparse as revealed by Legrand et al., adherence to standard and additional hospital hygiene guidelines in psychiatry departments must be improved, especially in emergency situations [7].

Most mental health-care departments appear to have set up infection control measures [8–18]. They also organised planning, coordination and measures that enabled them to provide psychiatric care in a restrictive environment. They were able to develop rapid, even innovative, strategies of adaptation. For example, the COVID-19 pandemic has accelerated the development of telepsychiatry and strengthened home care and liaison psychiatry [9, 19, 20].

The collection and evaluation of these organisational measures and strategies during the COVID-19 crisis appear important for understanding the course of the epidemic [21]. This collection needs to take place at local, national, and international levels. Currently, very few studies are available about the organisational response of psychiatric facilities to this pandemic [22–25].

Our objective was to assess the organisation by psychiatric facilities in France of their response to COVID-19 during the first wave which lasted from 5 March 2020 to 6 June 2020, while considering their timelines and their sector (public/private for-profit, private non-profit).

## Methods

### Timeline of the publication of the principal guidelines about the epidemic in France (see additional file 1)

#### Organisation of psychiatric care in France (see additional file 2)

##### Constitution of a structured audit grid

The audit grid was elaborated specifically for the study. To identify the different organisational measures taken and changes made in psychiatric facilities, a working group of seven professionals with diverse jobs and expertise (psychiatrists, psychiatric nurses, physician-hygienist, nurse-hygienist, pharmacists, a quality/risk management supervisor, and administrative staff) was set up to identify the recommendations published in their fields of expertise. We conducted a review of the international literature for this purpose. It enabled us to compile references for the principal publications related to opinions, recommendations and guidelines, operational feedback, and scientific studies of the organisational changes adopted by psychiatric hospitals during this period. The literature review was performed on PubMed, by querying the following key words: “COVID-19”, “psychiatry”, “mental health”, and “organisation”. A total of 207 articles were included in the literature review. Other bibliographic sources were also consulted to identify pertinent information, including but not limited to the World Health Organization (WHO), the French national authority for health (HAS), the HCSP, and various professional societies (SFHH, the French microbiology society, and French-speaking society of clinical nutrition and metabolism). An additional 100 references were included to assess the items of the audit grid. For each recommendation, we recorded its topic, its title, publication date, and recommended implementation date of the measure, if different; the organisational items judged important by the experts were extracted.

The working group identified 48 important items grouped in 4 major themes identified at the end of this work: general planning and coordination of the crisis management (10 items), specific measures associated with patient management and with their families in psychiatry departments (13 items), hospital hygiene and epidemic control measures (13 items), and management of human resources (5 items). In addition, 7 items were added to identify the profile of the persons audited (3 items) and the impact of COVID-19 on the facilities: number of patients with COVID-19, number of hospital staff infected by it, and number of deaths — all during the first wave (4 items). When it was considered relevant for a particular item, we included the date that this measure or action was added.

The project team, with support from the working group, selected and finalised the 48 items included for the survey questionnaire and the elaboration of an interview guide for the investigators. Table 1 synthesises the items considered in the audit grid and the type of response expected.

**Table 1:** Synthesis of items covered in the audit grid and expected type of response

Items	Type of response
*Profile of the individuals audited*	
Function of the person interviewed	Open
Membership in the crisis management group	Closed yes/no
Function in the crisis management group	Open
*Impact of the first wave of COVID-19*	
Number of patients with COVID-19 at or after admission during the first wave (March to early May 2020)	Categorical (0; [0–20[; [20–50[; 50 or more)
Number of staff members with COVID-19 during the first wave (March to early May 2020)	Continuous
Number of deaths due to COVID-19	Continuous
*Planning and coordination of crisis management*	
Activation of the crisis plan (“plan blanc”) and of the crisis management group	Date
A territorial partnership in mental health set up with a for-profit private, public, or private non-profit establishment	Closed yes/no
Bed management system set up	Closed yes/no
Availability of medical equipment at the start of the epidemic: blood pressure monitors, pulse oximeters, thermometers, semiautomatic defibrillator, suction aspirators, oxygen bottles, special steps taken to increase the stock of this equipment	Closed yes/no
Real-time inventory management system for personal protective equipment set up	Closed yes/no
Presence of occupational physician in the crisis management group	Categorical (Never/Rarely/Often/Very often/ Always)
Presence of staff representatives in the crisis management group	Closed yes/no
Frequency of information to staff representatives	Categorical (Never/Rarely/Often/Very often/ Always)
Frequency of information to user/patient representative	Categorical (in real time/daily/twice a week/ once a week/every two weeks/less often)
In a research study about COVID-19	Closed yes/no
*Specific measures related to the management of patients in psychiatry departments and their families*	
Reduction/adaptation of activity during lockdown period: full-time hospitalisation, day hospitalisation, outpatient consultations, CATTP activities, activity therapy, psychosocial rehabilitation activity, home care/visits	Categorical (Completely maintained/ Partially maintained/Closed/ Not concerned)
Specific activities initiated in-person consultations with adherence to barrier measures, telepsychiatry (video conferencing), telephone consultation, home visits with barrier measures	Closed yes/no
Maintenance of some activities that are part of psychiatric support: psychological follow-up, social support	Closed yes/no
Update of provisions to ensure the rights of patients, freedom of movement, protection of the dignity of hospitalised persons, organisation of hearings in front of the judge deciding on the liberty or detention of patients hospitalized without their consent, continuity of follow-up of patients obliged or mandated to attend psychiatric care	Closed yes/no Categorical (in person/videoconference/ judge decides alone, based on their own file, /other (specify)/not concerned)
Staff assigned for in-person or telephone availability for patient follow-up	Closed yes/no
Formalisation of a list of drugs at special risk with COVID-19	Closed yes/no
specific procedures for – food services, − laundry, − mail, − patient transport,	Closed yes/no
Establishment officially listed as admitting COVID-19/ opening of units exclusively for COVID-19	Closed yes/no
Formal official protocol for operation of COVID-19 units opened	Closed yes/no
Distribution of written instructions to patients to explain the barrier measures	Closed yes/no
"Listening services” for patients and their families	Closed yes/no
Maintenance of in-person family/friend visits and establishment of alternative means of communication	Closed yes/no
Remote follow-up for carers	Closed yes/no
Innovative arrangements	Closed yes/no, details
*Hospital hygiene and epidemic control measurement*	
Designation of an expert responsible for infection vigilance	Categorical (designated before the epidemic/ designated during the epidemic/not designated)
Advice sought from the EOH	Closed yes/no
Shortages of medical equipment	Categorical (surgical mask, FFP2 masks, gloves, smocks, detergents, disinfectant, disinfectant wipes, other) then closed yes/no
Systematic screening for signs suggestive of COVID-19 by a somatic physician at patient admission	Closed yes/no
Systematic testing for COVID-19 at admission	Closed yes/no
Established a specific procedure for patients with confirmed or suspected COVID	Closed yes/no
Screening at admission and specific follow-up of patients with risk factors for severe COVID-19	Closed yes/no // open (frequency)
Dedicated channel of care for the persons at risk	Closed yes/no
Specific programme to educate patients about barrier measures and social distancing	Closed yes/no // Categorical (< 10; [10–50[; [50–100[; [100–250[; [250–500[; > 500)
Specific training for professionals about additional precautions beyond hospital hygiene in the management of patients with COVID-19	Closed yes/no // Categorical (< 10; [10–50[; [50–100[; [100–250[; [250–500[; > 500)
Training some staff members to take nasopharyngeal samples for RT-PCR testing	Closed yes/no
Procedure for specific follow-up of risk of infection among staff (monitoring symptoms, seeing the occupational physician, nasopharyngeal tests, etc.)	Closed yes/no
Procedure for the management of persons who died with COVID-19	Closed yes/no
*Human resource management*	
Staff attendance chart, and chart of persons who can be called on if needed	Closed yes/no
human resources management enabling psychological care for staff (QoL at work plan), − telephone listening services for care providers	Closed yes/no // open
Organisational models to support and protect the health care staff and enable flexibility in staffing	Closed yes/no
Work at home (telecommuting) for some occupational categories	Open (type of category), Categorical ([0–25%[; [25–50%[; [50–75%[; [75–100%]
Study of the potential financial impact	Closed yes/no

### Study scope

Observational cross sectional quantitative study was performed (Evaluation Organisationnelle des établissements Psychiatriques: EvOlu’Psy study). The study covered all facilities in metropolitan France with a capacity for full-time, that is, inpatient psychiatric hospitalisation of adults (16 years and older) that exceeded the 3rd decile according to the annual establishment statistical survey (SAE) conducted in 2018. This administrative survey is mandatory and exhaustive; the ministry of health’s department of research studies, evaluation and statistics conducts it annually among all health-care facilities, public and private, in France [26]. The study base thus comprises all 331 establishments in metropolitan France with a capacity of 56.6 beds for full-time inpatient adult psychiatric hospitalisation, that is, 70% of the facilities in metropolitan France that provide this type of hospitalisation, according to the 2018 SAE survey [27]. This bed threshold was chosen because it enabled us to exclude excessively specific organisational models, while covering 70% of all facilities providing full-time inpatient psychiatric care to adults, including a large number of medium-sized public and private non-profit institutions.

The sampling plan was designed to optimise the representativeness of the situations experienced by the facilities while taking into account the survey resources available for the study. Our methodology aimed to obtain an empirical sample of responses, representative according to the quota method, based on the status of the facility (public, private non-profit, private for-profit).

### Audit

The initial survey questionnaire was pilot-tested in the planned survey conditions: the investigator entered responses during a telephone conversation with a qualified staff member working on the respondent facility’s crisis management, after this interlocutor had received an interview guide to enable him/her to know what the questions would be and thus prepare responses. Three establishments, included in the sampling base and known to the investigators (1 public, 1 private non-profit, 1 private for-profit), participated in the test. By administering the pilot-test under the planned conditions, we were able to assess its feasibility to be done in a fixed amount of time and the usability of the responses.

A survey team composed of one coordinator and 6 investigators, trained specifically for this survey, was recruited to conduct the initial contact and appointment scheduling. To ensure the rigour and reliability of this survey campaign and the responses obtained, the investigators underwent an initial 2-h training about the survey context and received standardised survey instructions, supporting documents, and each question in the interview guide. The project team also accompanied each investigator for their first interview. In addition, all investigators received a consolidated listing of information about contacts for all establishments in the sampling base, as well as a set of information relative to the survey, including the emails, telephone discourse for approaches and reminders, and the interview guide for the questionnaire, with an introduction explaining the survey objectives and procedures.

By the end of June 2020, all 331 establishments in the sampling base had been contacted by one of the 6 investigators, by email and telephone, and telephone appointments had been scheduled. At the appointment, the investigator entered the responses in the data collection tool, which was based on Qualtrics software (Qualtrics 2020©, Provo, UT, USA) and preset according to preparatory data collection work conducted in advance by the respondent facility with the interview guide. Interviews were performed in french between the last week of June 2020 until first week of September 2020.

Each establishment received regular email and telephone interviews until we obtained at least the number of appointments planned, by substrata. All appointments made took place, even after the objective of the relevant substrata had been attained. The responses above the objective were recorded to strengthen the reliability of the survey results for these substrata.

All methods were carried out in accordance with STROBE checklist.

### Statistical analysis

The descriptive data about the questionnaire items are presented as numbers and percentages. Facilities were compared for each item by status. The comparisons used a Chi-2 test or Fisher’s exact test, when necessary, because of the small numbers. All of the analyses were performed with SAS software (SAS v9.4, SAS Institute, Inc., Cary, NC, USA). The results concerning the measures taken were categorised to facilitate their analysis. Results ranging from 80 to 100% were judged very satisfactory, from less than 80 to 70% satisfactory, from less than 70 to 60% inadequate, and results less than 60% very inadequate.

## Results

Of the 331 establishments contacted, 94 (28.4%) agreed to respond to the survey questionnaire, administered by telephone by a trained investigator to guarantee the quality of the data entry; 29 (8.7%) refused to participate, and 208 (62.8%) did not respond at all.

### Establishment characteristics

The response rate from the public sector was 25.9% (*n* = 43), from the private non-profit sector 44.2% (*n* = 19), and from the private sector for-profit 26.2% (*n* = 32).

The distribution of facilities by number of beds was as follows: 26 facilities reported fewer than 100 beds, 28 from 100 to 200 beds, 27 from 201 to 500, 11 from 501 to 1000, and one more than 1000 beds. This number of beds includes all of the hospital beds in the establishment and not only its psychiatry beds.

### Audit results

#### Profile of the individuals audited

The profile of the people responding to the audit (*n* = 93) was categorised by their profession and/or the department they worked in: administration (41.94%, quality/risk management (22.58%), physician (17.20%), senior health manager (10.75%) and management of care (7.53%). In all, 91.49% (*n* = 86) of those audited reported that they were members of the hospital’s crisis management group.

#### Impact of COVID-19 on the facilities audited

The psychiatry departments of 47.90% (*n* = 45) of the facilities had admitted patients with COVID-19: 23.40% from 1 to 5 patients, 10.64% from 6 to 10, 3.19% from 11 to 15 and 10.64% more than 15 patients. The psychiatry departments of the facilities surveyed reported a total of 1987 patients with COVID during the first wave.

Among the establishments responding to the question about staff with COVID-19 during the first wave (*n* = 75), 41.33% reported none, 30.67% from 1 to 5, 1.33% from 6 to 10 and 26.67% more than 10. Overall, the respondents reported 881 staff members with COVID-19 during the first wave. Among the establishments that answered the question about the number of staff deaths (*n* = 83), 97.6% reported none, and 2.4% reported one or more. Overall, 3 staff deaths were reported.

#### Planning and coordination of crisis management

Partnerships were set up: 42.55% (*n* = 40) of the facilities reported setting up a partnership with a public institution, 8.51% (n = 8) with a private non-profit facility, and 22.34% (*n* = 21) with a private for-profit facility.

A bed management system was set up in 91.5% (*n* = 86) of the establishments surveyed.

Adequate quantities of the following types of medical materials were reported (percentage of affirmative responses in parentheses): blood pressure monitors (92.6%, *n* = 87), pulse oximeters (89.4%, *n* = 84), thermometers (57.5%, *n* = 54), single-use tips for thermometers (54.3%, *n* = 51), semiautomatic defibrillators (94.7%, *n* = 89), suction aspirators (90.4%, *n* = 85), oxygen bottles (89.4%, n = 84), and oxygen concentrators (83%, *n* = 78). A real time inventory management system was set up in 91.5% (*n* = 86) of the responding establishments.

Participation in the crisis management group varied: in 26.83% (*n* = 25) of establishments, the occupational physician was often or always at these meetings. In 51.06% (*n* = 48) of establishments, staff representatives participated in these crisis management meetings: often in 22.3% (*n* = 21), always in 19.1% (*n* = 18), and rarely in 9.6% (*n* = 9). Information about the situation during the crisis period was transmitted to staff representatives in real time in 27.7% (*n* = 26) of establishments, daily in 17% (*n* = 16), twice weekly in 9.6% (n = 9), weekly in 21.3% (*n* = 20), and less than once a week in 24.5% (*n* = 23).

The frequencies at which information was provided to patients and families by the establishment were: real time 8.5% (*n* = 8), daily 4.3% (*n* = 4), twice a week 4.3% (n = 4), weekly 12.8% (*n* = 12), and less than once a week 70.2% (*n* = 66).

COVID-19-related clinical trials were underway in 7.5% (*n* = 7) of the facilities.

#### Specific measures related to the management of patients in psychiatry departments and their families

Full-time inpatient hospitalisation was completely or partially maintained by 94.7% (*n* = 89) of facilities. Among those providing day hospitalisation, 96.39% (*n* = 80) closed or partially maintained it. CMP (outpatient consultations) activities were completely or partially maintained by 90% (*n* = 54), while CATTP (part-time therapeutic outpatient) activities were closed or partially maintained by 96.43% (n = 54), activity therapy (e.g., art or music) by 87.5% (*n* = 49), and psychosocial rehabilitation activities (outpatient) by 86.21% (*n* = 50). In 86.89% (*n* = 53) of responding establishments, home care and home visits were completely or partially maintained.

In-person consultations continued in 75.5% (*n* = 71) of establishments, with barrier measures applied. Telepsychiatry (video psychiatric consultations) were used in 74.5% (*n* = 70) of establishments and telephone consultations in 92.6% (*n* = 87). Home care was set up or continued in 54.26% (*n* = 51) of the facilities, 90.1% (*n* = 73) continued social support, 96.5% (*n* = 83) psychological support, and 79.8% (*n* = 75) set up (or already had) a staff member on duty in person or by telephone to provide patient follow-up.

Specific measures concerning respect for patients’ rights included updates of their procedures on the following themes, reported by 58% (*n* = 55) of establishments: freedom of movement (98.2% of these establishments, *n* = 54) and the dignity of hospitalized persons (81.5%, *n* = 44).

Some institutions (*n* = 51) providing care without consent reported that the civil hearings by a judge to determine the need for detention did not take place: 45.1% (*n* = 23) reported that the case was handled by the court based on the file only, 39.2% (*n* = 20) that it took place by videoconference, and 15.7% (*n* = 8) that all parties were present. Finally, 70.4% (*n* = 38) of the establishments receiving patients required or mandated by the courts to receive care reported that the patient and the court remained in contact.

Among the facilities responding to the audit, 54.3% (*n* = 51) were identified as reference hospitals available to receive patients with COVID-19 requiring psychiatric care, and 79.8% (*n* = 75) reported they had opened a unit intended to care for psychiatric patients with this disease. Among these, 86.8% (*n* = 66) reported they had developed and formalised a written protocol for the operation of this unit. Moreover, 36.2% (*n* = 34) had formalised a list of drugs at special risk with COVID-19.

Various helplines and hotlines (by telephone or internet) were set up to enable patients to express feelings, feel listened to, or state their needs or complaints by 67% (*n* = 63) of the facilities, and similar “listening services” for patients’ families were set up by 54.3% (*n* = 51). These arrangements were always available for patients in 54% (*n* = 34) of these establishments and for their families in 49% (*n* = 25).

Different policies were reported for family visits: 37.2% (*n* = 35) said visits were available in exceptional circumstances (worsening or precarity of the patient’s health), 3.2% (n = 3) had completely maintained in-person visits, while 59.6% (*n* = 56) had totally stopped them. Among the latter, 85.7% (*n* = 81) reported that they had replaced physical visits by alternative forms of contact. In addition, 47.9% (*n* = 45) of the facilities reported that they had arranged follow-up for caregivers by telephone and 26.6% (*n* = 25) by videoconference.

Logistic organization had been modified by specific procedures for food services (91.5%, *n* = 86), laundry (66%, *n* = 62), mail (47.9%, n = 45), and patient transport (54.2%, *n* = 51).

In all, 43.6% (*n* = 41) of facilities reported they had developed innovative arrangements during the first wave.

#### Hospital hygiene and epidemic control measures

Among respondents, 68.8% (*n* = 64) reported that they had designated an expert to be responsible for “infection vigilance”, that is, for monitoring, reporting and responding to infections, before the pandemic, and 95.7% (*n* = 90) had sought advice from their operational hygiene team (EOH).

Facilities reported that the following types of personal protective equipment and cleaning products had not been limited or out of stock (percentage so reporting in parentheses):

- Surgical masks (25.53%, *n* = 24),
- Smocks (29.79%, *n* = 28),
- FFP2 masks (43.62%, *n* = 41),
- Single-use non-sterile gloves (63.83%, *n* = 60),
- Detergent and disinfectant sprays (62.37%, *n* = 58),
- Disinfectant wipes (64.13%, *n* = 59),
- Detergents (69.89%, *n* = 65).

Nearly half the establishments (48.9%, *n* = 46) reported they had set up specific training programmes for patients about barrier measures and social distancing. Among them, 43.5% (*n* = 20) reported that fewer than 100 patients had done the programme, 30.4% (*n* = 14) from 100 to 250, 13% (*n* = 6) from 251 to 500, and 13% (n = 6) more than 500. Also among them, 95.7% (*n* = 44) reported that these programmes were still underway at the time of the survey. Overall, 86.2% (*n* = 81) reported having created written instructions for patients to explain the barrier measures.

Every facility had developed a written procedure for specific hygiene at admission of any patient with suspected or confirmed COVID-19. While 81.9% (*n* = 77) had initiated systematic checking for clinical signs suggestive of COVID-19 at admission, 49% (*n* = 46) had not launched a procedure of routine testing for COVID-19 by RT-PCR from nasopharyngeal samples.

Overall, 74.5% (*n* = 70) had set up a dedicated channel of care for patients at risk of severe COVID-19, and 52.1% (*n* = 49) a system for routine screening at admission for these risk factors.

Moreover, 84% (*n* = 79) reported they had set up staff training about additional precautions beyond standard hospital hygiene for COVID-19. Among them, 54.4% (*n* = 43) had trained fewer than 100 staff members, 19% (*n* = 15) between 100 and 250, 12.7% (*n* = 10) between 251 and 500, and 12.7% (n = 10) more than 500.

Staff training in taking nasopharyngeal samples was reported by 78.7% (*n* = 74).

In addition, 87.2% (*n* = 82) reported setting up one or more procedures for monitoring infectious risks among personnel, and 55.3% (*n* = 52) involved the occupational physician in this procedure.

According to 79.79% (*n* = 75) of the facilities, they had developed procedures for the management of the bodies of persons who died with COVID-19.

**Fig. 1:**
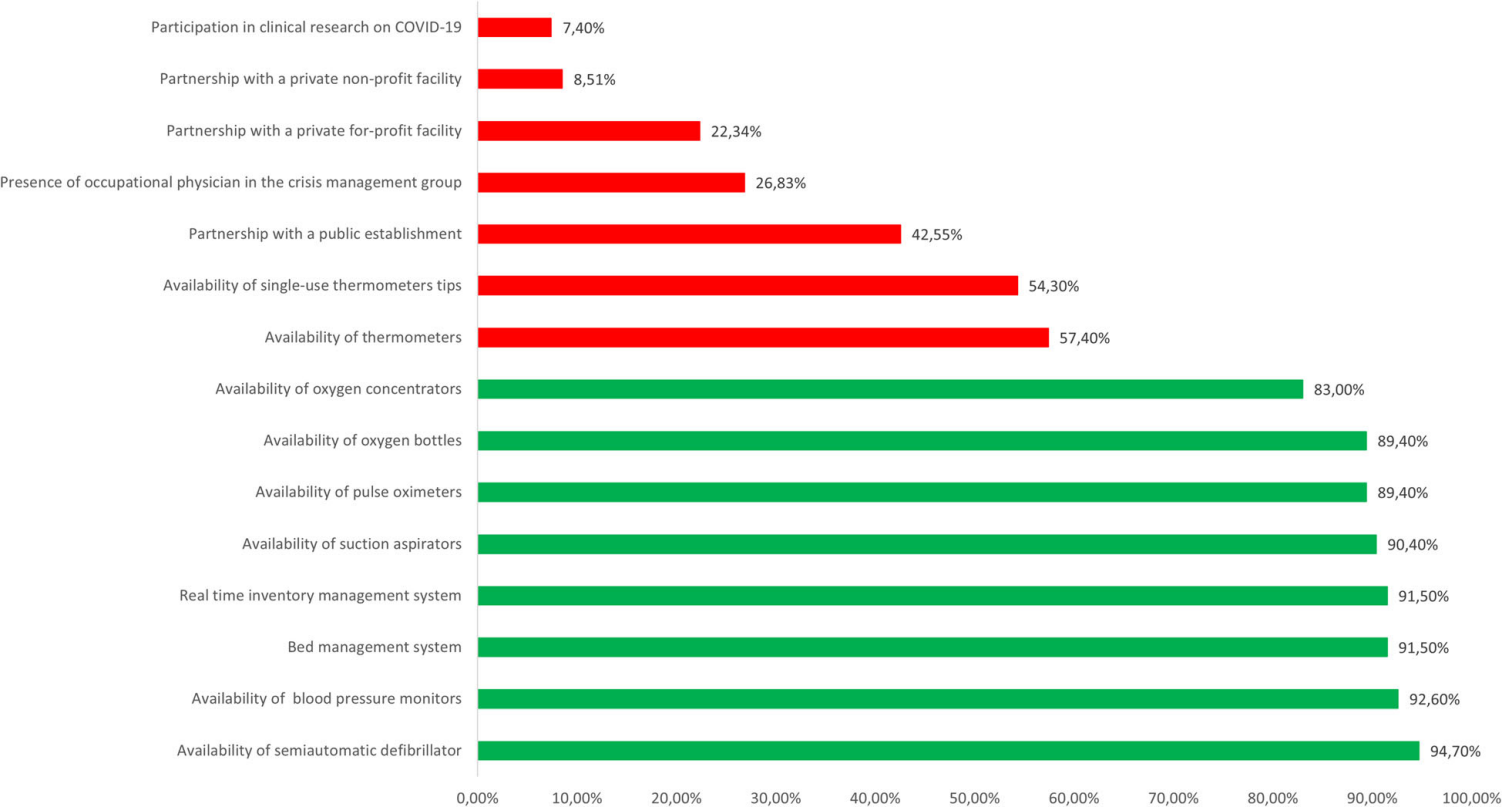
Results of the principal responses about the organisational responses of French psychiatric facilities, during the first wave of COVID-19, categorized according to the expected response level: Planning and coordination of crisis management

Figure 1 presents the results categorised by level of response.

#### Human resource management

Most (92.6%, *n* = 87) facilities had attendance charts as well as charts of the staff who could be called in if necessary. Measures enabling psychological care for staff were set up at 93.6% (*n* = 88) of establishments, and “listening services” for the health care workers at 85.23% (*n* = 75). Organisational models were implemented by 67% (*n* = 63) to support and protect the health care staff and enable flexibility in staffing. Telework (working from home) was set up for some occupational categories in 90.4% (*n* = 83) of the establishments. Finally, 35.1% (*n* = 33) had begun studies of the potential financial impact of this pandemic.

### Results of these measures by date

#### Planning and coordination of crisis management

The emergency “plans blancs” were activated in 36.2% (n = 33) of establishments on 13 March 2020; 72.3% (*n* = 68) had set up a COVID-19 crisis management group before that date.

Among responding facilities with a bed management system (*n* = 73) for continuous monitoring of the beds available, the system was operational before 22 March in 95.9%.

#### Specific measures related to the management of patients in psychiatry departments and their families (Fig. 2)

Dates by which various measures adapting specific activities were implemented in licensed psychiatry facilities are summarised:

- Full-time hospitalisation (*n* = 83): 83.1% had implemented these measures before 17 March 2020
- Day hospitals (*n* = 81): 87.7% before 23 March, 70.3% before 17 March
- outpatient consultations (CMP) (*n* = 58): 91.4% before 23 March, 79.3% before 17 March
- CATTP (*n* = 56): 92.9% before 23 March, 78.6% before 17 March
- Activity therapy (*n* = 54): 92.6% before 23 March, 77.8% before 17 March
- Psychosocial rehabilitation (*n* = 55): 92.7% before 23 March, 78.2% before 17 March
- Home visits (n = 58): 89.7% before 23 March, 77.6% before 17 March.

**Fig. 2:**
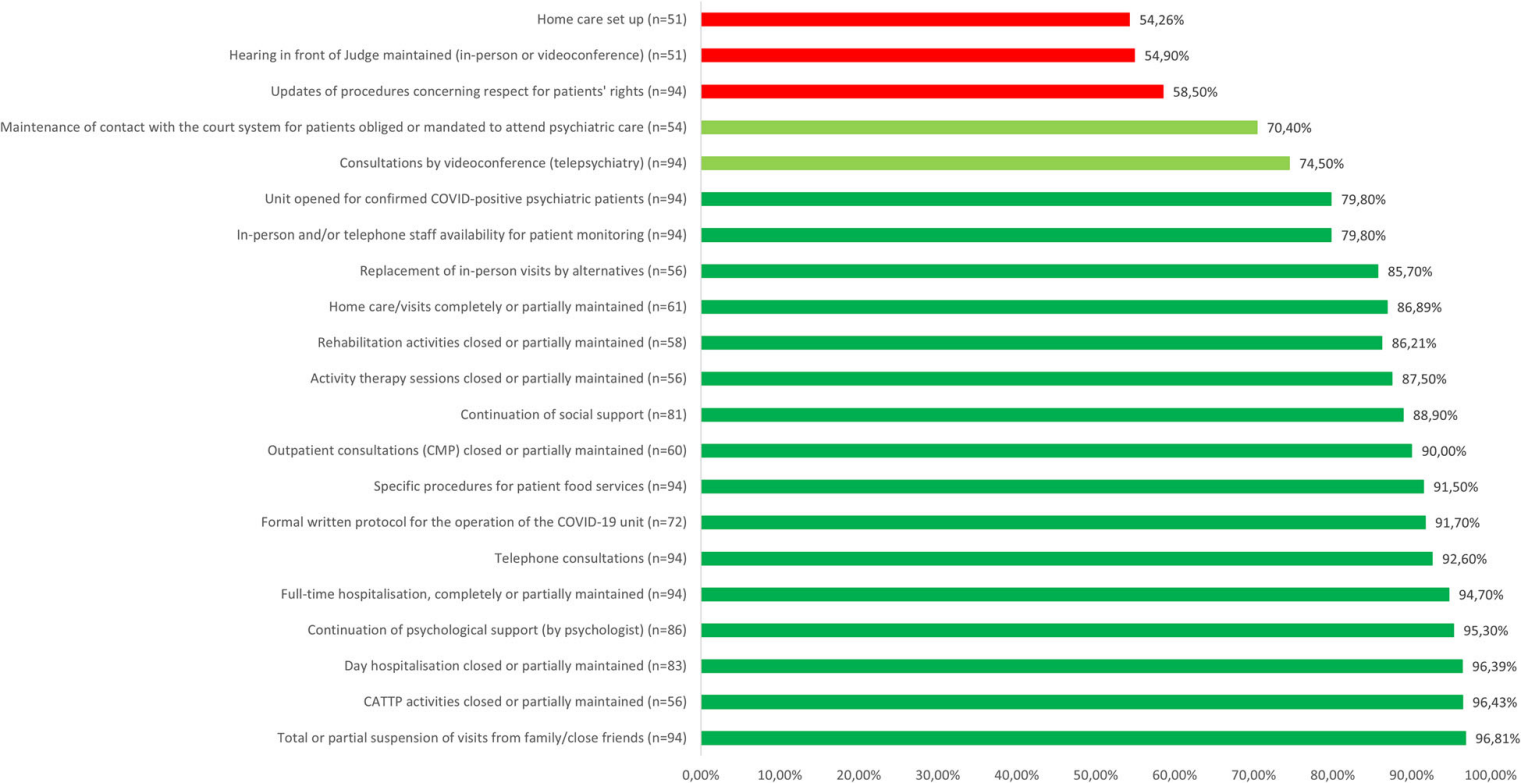
Results of the principal responses about the organisational responses of French psychiatric facilities, during the first wave of COVID-19, categorized according to the expected response level: Specific measures related to the management of patients in psychiatric departments and their families

Among the 70 establishments setting up in-person consultations with adherence to barrier measures, 28.6% were in place before 6 March, and 67.1% between 6 March and 22 March. Of the 87 facilities that responded that they had set up telephone consultations among the respondents, 90.8% had put them into place before 22 March. Of the 50 respondents organising home care, 88% had set it up before 17 March.

In all, 73 units planned to set up units for psychiatric patients with COVID-19; 64.4% had opened them before 22 March, and 35.6% opened them afterwards. Among the 64 units that responded, 60.9% had formalised a protocol for the operation of a COVID-19 unit and had opened it before 22 March.

Of the 68 units reporting that they had planned to set up teleconsultations (mostly video) for patients, 63.2% had done so before 16 March, 14.7% between 16 and 22 March, and 22.1% after that date.

Specific measures for food services were reported by 84 establishments; 95.2% of them reported implementing them before 23 March. Among the 51 respondents reporting specific measures for patient transport, 88.2% had set them up before 22 March.

In all, “listening services” for patients were reported by 56 respondents, 67.9% of which had put them in place before 22 March.

Suspension of family visits was reported by 89 establishments, 92.1% of them before 22 March. At the same time, 46 facilities reported setting up alternatives to in-person visits, 84.8% of them before 22 March.

#### Hospital hygiene and epidemic control measures (Fig. 3)

Of the 87 responding that they had sought advice from their reference hygiene team (EOH), 64.4% had done so before 13 March and 12.6% before 6 March.

**Fig. 3:**
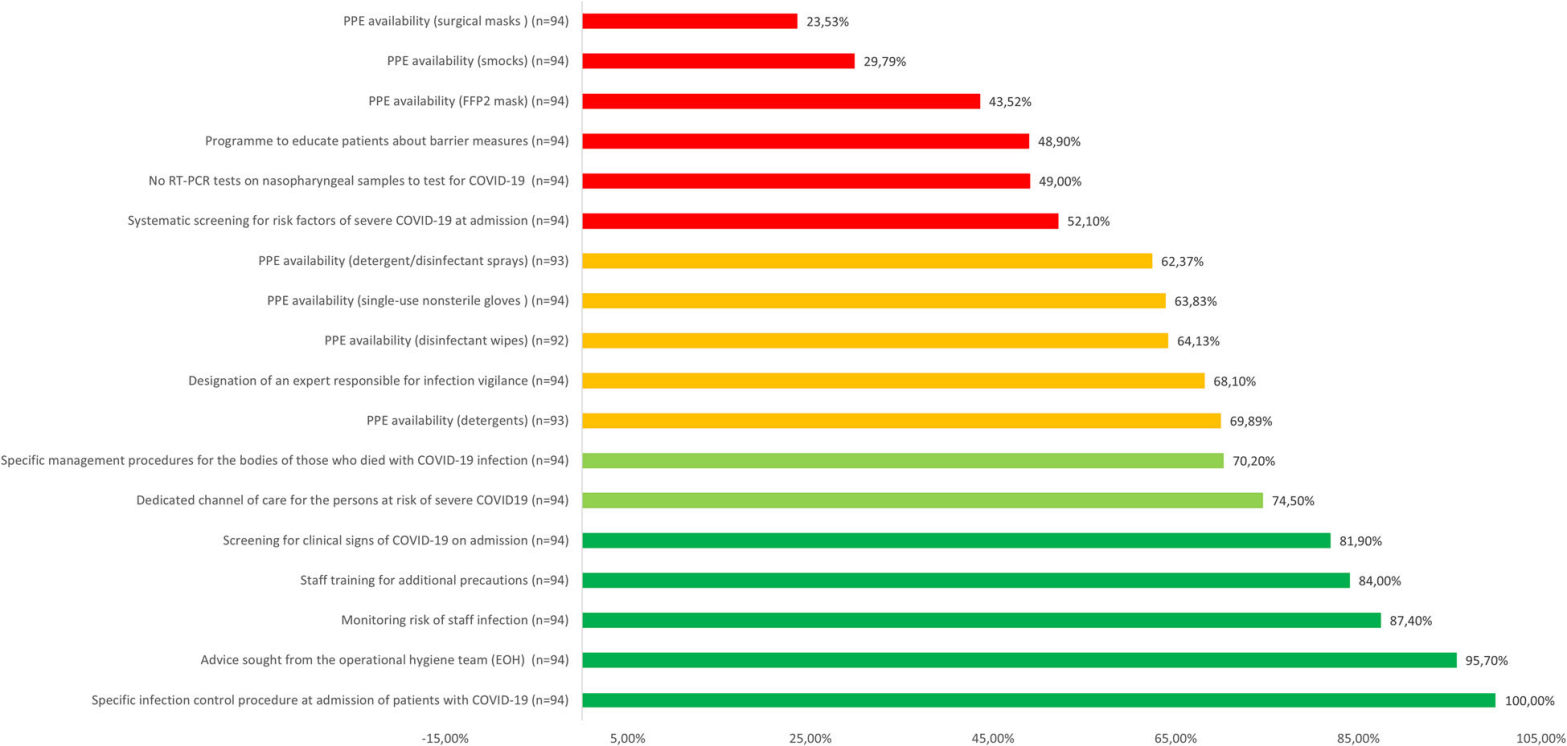
Results of the principal responses about the organisational responses of French psychiatric facilities, during the first wave of COVID-19, categorized according to the expected response level: Hospital hygiene and epidemic control measures. Very satisfactory . Satisfactory . Inadequate . Very Inadequate

Ninety-one establishments reported setting up a specific infection control procedure for patients with suspected, possible, or confirmed SARS-CoV-2 infections, 6.6% before 6 March, 34.1% from 6 to 14 March, and 59.3% after 14 March.

Implementation of screening for patients at risk for severe COVID-19 was reported by 46 facilities, 71.7% before 15 March, while 69 had set up dedicated care channels for these at-risk patients, 78.3% of them before that date.

Of the 76 hospitals that set up staff training specifically about additional precautions, 14.5% had done so before 6 March and 40.8% between 6 and 14 March.

Forty-five reported setting up educational programmes for patients about barrier measures and social distancing, 62.2% before 22 March.

Specific infection control procedures for the management of the body of patients who died with SARS-CoV-2 infections were instituted by 73 establishments, 6.89% of them before 6 March.

### Results by facility status

#### Profile of the individuals audited

The individual respondents from the public sector participated significantly less often in the crisis management group than those from the other establishments (*p* = 0.007).

#### Impact of COVID-19 on the facilities audited

The private non-profit facilities reported admitting significantly more patients with COVID-19 in their psychiatry departments (more than 15 patients) than the other establishments (*p* = 0.0002).

The private for-profit facilities reported significantly more often than the other groups (*p* < 0.0001) that they had no staff cases of COVID-19.

#### Planning and coordination of crisis management

Private establishments reported that they had activated their crisis plans before the national activation significantly more often than the public facilities (*p* = 0.0486). The private non-profit facilities reported setting up territorial partnerships with other private non-profit facilities significantly more often than the other institutions (*p* = 0.0197).

Public institutions reported that the occupational physician was always, often, or very often present at crisis management group meetings significantly more often than the others (*p* < 0.0001). They also reported significantly less often that they had set up a real time inventory management system for personal protective equipment (*p* = 0.03). They were also the only type of institution to set up scientific studies of COVID-19 during the first wave.

#### Specific measures related to the management of patients in psychiatry departments and their families

Private non-profit facilities reported significantly more often than the other groups (*p* = 0.04) that they had closed (or partially closed) their outpatient psychosocial rehabilitation activities. The public establishments reported setting up in-person consultations with adherence to barrier measures significantly more often than private facilities (*p* = 0.008). They also reported setting up telephone consultations significantly more often (*p* = 0.005). Private facilities, on the other hand, reported setting up home visits with adherence to barrier measures significantly less often than their public counterparts (*p* < 0.0001). Private non-profit facilities reported maintaining their social support more often than the other types of facilities (*p* = 0.01).

The public institutions stated that they had updated their provisions about ensuring patients’ rights significantly more often than private facilities (*p* = 0.03). They had also set up “listening services” for patients significantly more often (p = 0.03). Finally, they were publicly listed as accepting patients with COVID-19 significantly more often than the private facilities (*p* < 0.0001) and, accordingly, they had set up significantly more COVID units (*p* = 0.003).

#### Hospital hygiene and epidemic control measures

The private for-profit facilities had sought advice from their EOH teams significantly less often than the others (*P* = 0.023) and reported significantly less frequent shortages of the following personal protective equipment: gloves (*P* = 0.01), smocks (*P* = 0.001), and detergent-disinfectant sprays (*P* = 0.02).

Public institutions, on the other hand, had set up staff training programs for nasopharyngeal sampling for RT-PCR testing significantly more often than private facilities (*p* = 0.01).

Private non-profit facilities had set up special procedures for managing the bodies of patients who died with COVID-19 more often than the others (*p* = 0.05).

#### Human resource management

Public institutions stated that they had set up “listening services” for health care providers significantly more often than the other types of establishments (*p* = 0.003). While private non-profit facilities reported that they had set up specific organisational models to support and protect the health care staff and facilitate flexibility significantly less often (*p* < 0.0001), they had set up studies of the potential financial impact of the pandemic significantly more often (*p* = 0.003).

The results concerning the measures taken by type of establishment are reported in Table 2.

**Table 2:** Results of the responses about the organisational responses of French psychiatric facilities according to their legal status

Items	Response expected	Total number 94 N %	Public 43 N %	Private non-profit 19 N %	Private 32 N%	p
*Profile of the individuals audited*						
Membership in the crisis management group	Yes	91.49	81.40	100	100	*0.0070*
*Impact of the first wave of COVID-19*						
Number of patients with COVID-19 at or after admission during the first wave (March to early May 2020)	> 15	10 10.64	6 13.95	4 21.05	0 0	*0.0002*
Number of staff members with COVID-19 during the first wave (March to early May 2020)	0	31 41.33	6 21.43	2 13.33	23 71.88	*<.0001*
*Planning and coordination of crisis management*						
Activation of the crisis plan ("plan blanc") before national activation	Yes	36.17	23.26	42.11	50	*0.0486*
Activation of the crisis management group before activation of the national "plan blanc"	Yes	64.89	55.81	84.21	65.63	*0.0965*
Territorial partnership in mental health with:						
- A private facility	Yes	22.34	23.26	21.05	21.88	*0.9788*
- A public institution	Yes	42.55	48.84	36.84	37.5	*0.5267*
- A private non-profit facility	Yes	8.51	9.3	21.05	0	*0.0197*
Bed management system set up	Yes	91.49	88.37	89.47	96.88	*0.4470*
Real time inventory management for personal protective equipment set up	Yes	91.49	83.72	94.74	100	*0.0252*
Availability of medical equipment at the start of the epidemic:						
- Blood pressure monitors,	Yes	92.55	88.37	100	93.75	*0.3524*
- Pulse oximeters,	Yes	89.36	83.72	100	90.63	*0.1536*
- Thermometers,	Yes	57.45	58.14	57.89	56.28	*0.9857*
- Thermometer tips and covers	Yes	54.26	53.49	57.89	53.13	*0.9380*
- Semiautomatic defibrillators,	Yes	94.68	93.02	94.74	96.88	*0.8450*
- Suction aspirators,	Yes	90.43	88.37	94.74	90.63	*0.9016*
- Oxygen bottles,	Yes	89.36	90.7	78.35	93.75	*0.3351*
- Oxygen concentrators	Yes	82.98	86.05	68.42	87.50	*0.1653*
- Special steps taken to increase the stocks of these types of medical equipment	Yes	63.83	67.44	78.95	50	*0.0919*
Presence of occupational physician in the crisis management group	At least often	26.83	46.15	14.29	6.90	*<.0001*
In a research study about COVID-19	Yes	7.45	100	0	0	*0.0104*
*Specific measures related to the management of patients in psychiatry departments and their families*						
Activity during the lockdown period:						
- Full-time hospitalisation,	Completely maintained/ partially maintained	94.68	93.02	100	93.75	*0.7083*
- Day hospitalisation,	Closed/partially	83 96.39	42 97.67	18 94.74	20 95.24	*0.7936*
- Consultation activity,	Completely maintained/ partially maintained	60 90	39 92.86	14 87.5	1 50	*0.1551*
- CATTP activities,		56	38	14	2	*1*
	Closed/partially	96.43	95	100	100	
- Activity therapy	Closed/partially	56 87.5	29 90.63	12 85.71	8 80	*0.0876*
- Psychosocial rehabilitation activity		58 86.21	28 90.32	16 94.12	6 60	*0.0431*
- Home care/visits	Closed/partially	61	37	13	3	*0.1597*
	Completely/partially	86.89	90.24	86.67	60	
Started specific activities						
- In-person consultations with adherence to barrier	Yes	75.53	86.05	84.21	56.25	*0.0075*
- measures,	Yes	74.47	74.42	84.21	68.75	*0.4726*
- Telepsychiatry,	Yes	92.55	100	94.74	81.25	*0.0054*
- Telephone consultations, Home visits with barrier measures	Yes	54.26	83.72	73.68	3.13	*<.0001*
Maintenance of some activities that are part of psychiatric support:						
- Psychological follow-up	Yes	86 96.51	41 100	18 94.74	24 92.31	*0.1387*
- Social support	Yes	81 90.12	39 94.87	19 100	23 73.91	*0.0120*
Staff assigned for in-person or telephone availability for patient follow-up	Yes	79.79	90.7	89.47	59.38	*0.0019*
Update of provisions to ensure patients' rights, freedom of movement, protection of the dignity of hospitalised persons, organisation of hearings in front of the judge deciding on the liberty or detention of patients hospitalized without their consent, continuity of monitoring of patients obliged or mandated to attend psychiatric care	Yes	58.06	50	11.11	38.89	*0.0319*
Establishment officially listed as admitting patients with COVID-19	Yes	54.26	79.07	52.63	21.88	*<.0001*
Units exclusively for patients with COVID-19	Yes	79.79	95.35	68.42	65.63	*0.0025*
Formal official protocol for operation of any COVID-19 unit opened	Yes	86.84	83.33	86.67	94.74	*0.5889*
Formalisation of a list of drugs at special risk with COVID-19	Yes	36.17	32.56	31.58	43.75	*0.5453*
Distribution of written instructions to patients to explain the barrier measures	Yes	86.17	88.37	78.95	87.5	*0.6307*
Specific procedures established for						
- Food services,	Yes	91.49	86.05	100	93.75	*0.1793*
- Laundry,	Yes	65.96	69.77	78.95	53.13	*0.1318*
- Mail,	Yes	47.87	48.84	42.11	50	*0.8490*
- Patient transport,	Yes	54.26	65.12	57.89	37.5	*0.0560*
"Listening services" for:						
- patients	Yes	67.02	79.07	68.42	50	*0.0297*
- their families	Yes	54.26	60.47	63.16	40.63	*0.1596*
Maintenance of in-person visits by family and close friends	No or rarely	96.81	100	84.21	100	*0.0072*
Alternative means of communication	Yes	85.71	92	85.71	79.17	*0.3732*
Remote follow-up for carers	Yes	100	100	100	100	*-*
- By telephone	Yes	47.87	60.47	42.11	34.38	*0.0699*
- By video meetings	Yes	25	27.91	42.11	15.63	*0.1135*
Innovative arrangements	Yes	43.62	46.51	57.89	31.25	*0.1563*
*Hospital hygiene and epidemic control measurement*						
Advice sought from the EOH	Yes	95.74	100	100	87.5	*0.0232*
Designation of an expert responsible for infection vigilance	Yes/present before the epidemic	68.82	71.43	68.42	65.63	*0.8664*
Shortages of personal protective equipment:						
- Surgical masks	No	25.53	25.58	21.05	37.5	*0.8548*
- FFP2 masks	No	43.62	51.16	31.58	40.63	*0.3276*
- Gloves	No	63.83	51.16	57.86	84.38	*0.0104*
- Smocks	No	29.79	20.93	10.53	53.13	*0.0013*
- Detergents	No	93 69.89	29 67.44	11 61.11	25 78.13	*0.4039*
- Detergent/disinfectant sprays	No	93 62.37	24 55.81	8 44.44	26 81.25	*0.0173*
- Disinfectant wipes	No	92 64.13	29 69.05	9 50	21 65.63	*0.3615*
Systematic screening for signs suggestive of COVID-19 by a somatic physician at patient admission	Yes	81.91	83.72	84.21	78.13	*0.7896*
Systematic testing for COVID-19 at admission	No	48.94	44.19	42.11	59.38	*0.3433*
Specific procedure for patients with confirmed or suspected COVID	Yes	100	100	100	100	*-*
Screening at admission and specific follow-up of patients with risk factors for severe COVID-19	Yes	52.13	39.53	57.89	65.63	*0.0699*
Dedicated channel of care for the persons at risk	Yes	74.47	76.74	68.42	75	*0.7837*
Specific programme educating patients about barrier measures and social distancing	Yes	48.94	44.19	52.63	53.13	*0.6988*
Specific training for professionals about additional precautions beyond hospital hygiene in the management of patients with COVID-19	Yes	84.04	88.37	84.21	78.13	*0.4875*
Training some staff members to take nasopharyngeal samples for RT-PCR testing	Yes	78.72	90.7	78.95	62.5	*0.0128*
Procedure for specific follow-up of risk of infection among staff (monitoring symptoms, seeing the occupational physician, nasopharyngeal tests, *etc*.)	Yes	87.23	90.70	73.68	90.63	*0.1976*
Procedure for the management of bodies of patients who died with COVID-19	Yes	79.79	86.05	89.47	65.63	*0.0467*
*Human resource management*						
Staff attendance chart, and chart of persons who can be called on if needed	Yes	92.55	93.02	94.74	90.63	*1*
Human resources management enabling psychological care for staff (QoL at work plan),	Yes	93.62	97.67	100	84.38	*0.0517*
- Telephone "listening services" for the health care workers	Yes	85.23	95.24	73.68	77.78	*0.0272*
Organisational models to support and protect the health care staff and enable flexibility in staffing	Yes	67.02	83.72	78.95	37.5	*<.0001*
Work at home for some occupational categories	Yes	90.43	93.02	94.74	84.38	*0.4234*
Study of the potential financial impact	Yes	35.11	30.23	68.42	21.88	*0.0032*

### Results by time of implementation and type of establishment

Private for-profit facilities set up crisis management groups earlier (between 6 and 13 March) than the other types of establishment (*p* = 0.03). Public institutions moved fastest to set up telephone consultations (between 14 and 16 March (p = 0.003), while the private facilities, both for-profit and non-profit, moved to reorganize their outpatient rehabilitation activities significantly earlier (*P* = 0.04) between 6 and 13 March. The private for-profit institutions moved fastest to make arrangements for “listening services” for patients (between 14 and 16 March (*p* = 0.03). Public institutions set up their programmes to educate patients about barrier measures and social distancing significantly earlier (p = 0.03) than the other types of institutions — between 14 and 16 March.

## Discussion

French psychiatric establishments on the whole were able to provide a necessary reorganisation of their management of patients and their families, regardless of facility status. Thus, day hospitalisation, CATTP activities, and psychosocial rehabilitation were reorganised toward either complete closure or maintenance of some activities. These results are consistent with those of a study from Italy, which found that the number of day hospitalisation days diminished by 78%, while those of day hospitalisation for psychosocial rehabilitation dropped by 85% [28]. This reorganisation of psychiatric activities took place very quickly, in many establishments before the French guidelines for psychiatric facilities were published on 23 March 2020. To our knowledge, there are no data in the literature with which we can compare this organisational timeline, but it is important to note that these French governmental guidelines specific for psychiatry appeared rather late — on 23 March although the national lockdown began on 17 March. Nonetheless, a very large majority of psychiatric facilities had made and implemented these measures before the lockdown. Implementation of home care, on the other hand, was very inadequate: only half of the facilities responding to this survey set it up during the first COVID-19 wave. We also note that specific guidelines for the management of patients with psychiatric diseases who were confined at home were published very late, on 2 April [29]. Facilities in the private for-profit sector set up these measures least often. These results are explained by the fact that home care is essentially managed in France by “public service” establishments; this type of care is not part of the culture of private for-profit facilities. On the other hand, these private for-profit facilities set up “listening services” more rapidly, probably to make up for their lesser use of home care.

The other measures considered very inadequate concerned essentially the ethical and legal aspects. Only 58.50% of the facilities reported that they had updated their procedures concerning the patients’ freedom of movement and the dignity of hospitalised people. Nonetheless, the restriction of liberties induced by the measures sometimes necessary to limit disease transmission (respiratory isolation measures) has raised again the fundamental question of the rights of patients in psychiatric hospitals [30]. Some psychiatric hospitals in China systematically isolated all new patients for 14 days before admitting them to specific hospital departments [31]. The COVID-19 crisis appears to have had a disproportionate effect on the loss of civil and political rights of persons with mental and cognitive disorders, which must nonetheless be considered absolute to guarantee respect of the person’s fundamental rights [32]. French public establishments appear to have done a better job of considering the issue of patients’ rights than the private facilities. No comparative data are currently available but this result is probably is explained by the fact that patients admitted without consent can only be admitted to establishments providing public service, which are principally public institutions.

Along the same lines, only 54.9% of the establishments responding to our survey reported that the hearings continued to take place in front of a judge to determine their detention (in-person hearings complying with barrier measures or videoconferences) for patients hospitalised in psychiatry departments without their consent. Although these hearings depend on the court system rather than the psychiatric hospitals, these results highlight once again the very inadequate consideration of these patients’ rights. On 27 March 2020, the controller general of places of deprivation of liberty alerted the ministry of health about several points concerning the rights of psychiatry inpatients and warned especially that most of the judges involved were not traveling to hospital sites and were ruling solely based on their own files [33].

The quality of the measures taken for hospital hygiene and epidemic control in the French psychiatric establishments during the first COVID-19 wave seems more mixed. Numerous actions in this domain appear to be inadequate or very inadequate. Thus we note that only 68.1% of the facilities had appointed an expert to be responsible for infection vigilance before the health crisis. Nonetheless, health monitoring in France, which includes infection vigilance, was instituted by Law n°98–535 dated 1 July 1998 to reinforce health surveillance and the safety of products intended for human use [34]. Infection vigilance is thus the set of specific measures of surveillance, prevention, and control of infections associated with care. It includes, in particular, the facility’s measures to combat health care-associated infections and its reporting of specific infections to the health authorities. The comprehensive and coordinated management of risks of health care-associated infections is also set forth in the law known as the Hospital, Patient, Health, and Territories Act, dated 21 July 2009, which specifies that these vigilance systems intended to ensure health security are among the responsibilities of each institution’s medical committee [35]. Moreover, even though 95.7% of the facilities reported seeking advice from their EOH during the first wave of the health crisis, only 64.4% had done so before the activation of the emergency “plan blanc”. Each French health care facility is nonetheless required to have an EOH, a hygiene operational team. The team is composed of experts in the management of the risk of infection of patients, professionals, and all persons who visit the facility. Among its missions, the EOH must initiate and coordinate the a priori management of infectious risks and is especially responsible for elaborating, disseminating, and setting up protocols in collaboration with clinical departments for the prevention of infections that may be associated with health care [36].

The first recommendation for hospital hygiene related to COVID-19 in France were published by the SFHH on 20 January 2020 and by the HCSP on 27 February [37, 38]. It thus seems surprising that the EOHs were not sought out for advice in this health crisis before 6 March. Similarly, although all establishments reported that they had set up specific infection control procedures for the admission of patients with COVID-19, more than half of them (59.3%) reported these were set up after 14 March, that is, more than 15 days after the HCSP recommendations on this topic were published. The procedures set up for the management of the bodies of deceased patients with COVID-19 were also late since 93.2% of the facilities who set them up did so after 6 March, and 31.5% after 23 March. Private non-profit facilities set up these procedures most often, perhaps because they also reported admitting more patients with COVID-19.

The results concerning personal protective equipment were judged very inadequate for surgical masks, smocks, and FFP2 masks, and inadequate for the other personal protective equipment. The shortage of personal protective equipment and especially masks was not unique to French psychiatric facilities. It affected all types of facilities and specialties, not only nationwide but around the world, starting at the onset of the first wave in China [39]. Although the implementation of systematic screening for signs of COVID-19 by the psychiatric facilities was very satisfactory, that of systematic screening of individuals with risk factors for severe COVID-19 was not; it was indeed very inadequate, taking place in barely half of the responding facilities (52.1%). This result reinforces the need to improve somatic management of patients with severe chronic psychiatric disorders, as underlined in the clinical practice guidelines in psychiatry issued in June 2015 and approved by the French national authority for health [40]. Finally, surprisingly, more than half of French psychiatric establishments set up routine testing for COVID-19 by nasopharyngeal samples tested by RT-PCR for the virus genome at admission, although no recommendation for this measure had been made for psychiatric facilities. The implementation of this type of measure appears to be associated with the strong desire of these establishments to control the virus and especially to protect their staff. That is, although they did not set up adequate measures to screen for patients at risk, the follow-up of the risk of infection among staff members was very satisfactory (87.4%) as was staff training in additional COVID-19 control precautions (84%). On the other hand, the implementation of an educational programme for patients about barrier measures was very inadequate — only 48.9%.

It thus appears necessary to promote hospital hygiene in psychiatry. To our knowledge, the literature about infection control measures in psychiatry is extremely sparse. A study conducted in 2019 about the practice of intramuscular injections in psychiatry departments suggests that standard hospital hygiene precautions require improvement in these departments, especially in emergency conditions [7]. Other studies on this subject are nonetheless necessary to confirm these results.

In terms of crisis management, in general, it should be noted that none of the different types of establishments collaborated very much during this epidemic wave. Only public institutions reported participating in clinical research on COVID-19, and the inclusion of the occupational physician in the crisis management group was very inadequate, although least poor in the public hospitals. To our knowledge, there are currently no data available in the literature about these organisational points and measures in psychiatric facilities during the first Covid-19 wave. We note that the facilities responding to our survey reported very inadequate availability of thermometers (available in only 57.4%), although these are essential in this epidemic situation.

The principal strength of our study is that it is the first to assess the organisation and measures taken in response to COVID-19 by French psychiatric facilities and their timeline in relation to the date that the principal national recommendations were issued and by the type of establishment. Our study sample is representative of psychiatric inpatient facilities in France. We sought to include 80 facilities and were able to include 94. Moreover, our results are exhaustive, with few missing data in the responses. Finally, it should be noted that a very high percentages of the survey respondents belonged to the establishment’s crisis management group; the information collected thus appears reliable. The limitations of our study are that the data collection is based on a telephone interview three to four months after the implementation of these measures. This survey is based on an internal audit without external evidence, such as documents and protocols. Nonetheless, we note that the respondents knew the questions in advance to enable them to collect the information to provide during the telephone interview.

## Conclusions

In conclusion, french psychiatric establishments on the whole were able to provide a necessary reorganisation of their management of patients and their families, regardless of facility status. Moreover, these facilities appear to have set up their responses quite rapidly, often earlier than the national recommendations on this topic. Patients’ rights nonetheless seem to have not received the attention they merited during the early pandemic period, and special and urgent attention to monitoring this aspect is essential to ensure that the individual liberties of psychiatric inpatients are respected in the future. Moreover, somatic management of patients with mental illness must absolutely be improved, as must the screening for risk factors for severe COVID-19, which are frequent in these patients.

Finally, the measures implemented for hospital hygiene for infection control in psychiatry during this first epidemic wave appeared inadequate, even very inadequate, or implemented too late. The promotion of hospital hygiene is essential.

## Supplementary Information

**Additional file 1.**

**Additional file 2.**

## Data Availability

The data that support the findings of this study are available from the corresponding author GL, upon reasonable request.
