# Supplementary material for: Organisation and timeline of measures in French psychiatric establishments during the first wave of the COVID-19 epidemic: EvOlu’Psy study
Source: BMC Psychiatry. 2021 Jun 2;21:284. doi: 10.1186/s12888-021-03293-0 (PMC8169419; doi:10.1186/s12888-021-03293-0)
Supplement: Supplementary file 1 — Additional file 1. [file 12888_2021_3293_MOESM1_ESM.docx]

**Additional file 1: Timeline of the publication of the principal guidelines about the epidemic in France**

In France, on 20 February 2020, the health ministry published the first volume of a methodological guide about the organisation to be set up to deal with the first epidemic wave of COVID-19, entitled "Preparation for the COVID-19 epidemic risk: Healthcare facilities, physicians in private practice, and medicosocial facilities" [1]. The emergency plan ("plan blanc") was activated in the hospitals of the regions most strongly affected on 6 March and enlarged to all hospitals in France on 13 March. This plan is intended to enable a healthcare facility to prepare immediately all of the resources it has available for the possible influx of patients or victims and thus cope with an exceptional health situation. On 16 March, the French government published the second volume of the methodological guide, entitled "Preparation for the COVID-19 epidemic phase" directed at the same groups [2]. The nationwide lockdown began on 17 March and continued until 11 May. The French government did not publish the first specific guidelines for licensed inpatient psychiatry establishments until 23 March [3].

The principal recommendations for specific management for infection control and hospital hygiene came from the high council on public health (HCSP) and the French society of hospital hygiene (SFHH). Accordingly, the SFHH published the first recommendations about the barrier measures (additional hospital hygiene precautions) to implement for the management of patients infected by COVID-19 on 28 January 2020; they were completed by the HCSP recommendation on 27 February [4, 5]. These guidelines were modified several times during the first wave. On 15 March, the HCSP published a document about issues related to screening patients at risk of severe COVID-19 and organising dedicated channels of care for them [6]. Finally, the HCSP published recommendations for the management of the body of deceased patients infected by SARS-CoV-2 virus on 27 February and modifications to them on 24 March [7, 8].

References :

1. Ministère des Solidarités et de la Santé. Préparation au risque épidémique Covid-19: Établissements de santé, Médecine de ville, Établissements médico-sociaux, Guide méthodologique. 2020. https://solidarites-sante.gouv.fr/IMG/pdf/guide_methodologique_covid-19-2.pdf.

2. Ministère des Solidarités et de la Santé. Préparation à la phase épidémique de Covid-19 : Établissements de santé, Médecine de ville, Établissements et services médico-sociaux, Guide méthodologique. 2020. https://solidarites-sante.gouv.fr/IMG/pdf/guide-covid-19-phase-epidemique-v15-16032020.pdf.

3. Ministère des Solidarités et de la Santé. Etablissements autorisés en psychiatrie : Consignes et recommandations applicables à l’organisation des prises en charge dans les services de psychiatrie et les établissements sanitaires autorisés en psychiatrie. 2020. https://s3-eu-west-1.amazonaws.com/static.hospimedia.fr/documents/206958/5146/Fiche_recommandations_Covid-19_psychiatrie.pdf?1584983108.

4. Société française d’Hygiène, Hospitalière. Avis relatif aux mesures d’hygiène pour la prise en charge d’un patient considéré comme cas suspect, possible ou confirmé d’infection à 2019-nCoV. 2020. https://www.sf2h.net/wp-content/uploads/2020/01/Avis-prise-en-charge-2019-nCo-28-01-2020.pdf.

5. Haut Conseil de la santé publique. Avis relatif au traitement du linge, au nettoyage d’un logement ou de la chambre d’hospitalisation d’un patient confirmé à SARS-CoV-2 et à la protection des personnels. 2020. https://www.hcsp.fr/Explore.cgi/Telecharger?NomFichier=hcspa20200218_corsarcovnetdeslocetprodesper.pdf.

6. Haut Conseil de la santé publique. Recommandations relatives à la prévention et à la prise en charge du COVID-19 chez les patients à risque de formes sévères. 2020. https://www.hcsp.fr/Explore.cgi/Telecharger?NomFichier=hcspa20200314_aprrlpelpecdcclprdfs.pdf.

7. Haut Conseil de la santé publique. Avis relatif à la prise en charge du corps d’un patient décédé infecté par le virus SARS-CoV-2. 2020. https://www.hcsp.fr/images/pdf3.gif.

8. Haut Conseil de la santé publique. Avis relatif à la prise en charge du corps d’un patient cas probable ou confirmé COVID-19. 2020. https://www.hcsp.fr/Explore.cgi/Telecharger?NomFichier=hcspa20200324_cosacoprenchducodunpaco.pdf.
