# Supplementary material for: Organisation and timeline of measures in French psychiatric establishments during the first wave of the COVID-19 epidemic: EvOlu’Psy study
Source: BMC Psychiatry. 2021 Jun 2;21:284. doi: 10.1186/s12888-021-03293-0 (PMC8169419; doi:10.1186/s12888-021-03293-0)
Supplement: Supplementary file 2 — Additional file 2. [file 12888_2021_3293_MOESM2_ESM.docx]

**Additional file 2: Organisation of psychiatric care in France**

The organization of psychiatric care in France is divided among three main types of providers:

- Public institutions (general or specialised hospitals offering inpatient care and medical-psychological consultations for outpatients),
- Private establishments participating in public service, administered by an association or mutual insurance company (healthcare facilities referred to as private of collective interest or private non-profit), and
- Private for-profit establishments (doctors' offices and psychiatric clinics).

The French hospital sector comprises 1,364 public establishments, divided into four subtypes:

- 178 regional hospital centres that provide the most specialised care to their region's population, as well as routine care for the local population including psychiatric services;
- 947 intermediate-category hospital centres, managing most short hospitalisations (medicine, surgery, psychiatry, obstetrics, and odontology), as well as care for the elderly;
- 95 hospital centres specialised in psychiatry;
- and 144 other public establishments, most of them providing long-term care.

Among the private hospital structures, there are 1,002 private for-profit clinics and 680 private non-profit facilities. Some of them are specialised in psychiatry.

In comparison with other medical disciplines, psychiatry has several specific characteristics:

- Few technical procedures
- Many outpatient facilities
- Management of recurrent and diversified patients.

Psychiatric care is essentially provided by public establishments, especially for outpatient psychiatric hospitalisation. Psychiatric care as a public service is provided in France by the public and private non-profit facilities. Each French district (*département*, France's basic administrative division) has been divided into geographic zones, referred to as "psychiatric sectors". The psychiatric sector simultaneously designates the geographic area but also the mental health care services available within its boundaries. A sector covers a territory of around 67,000 inhabitants [1, 2].

As a general rule, this psychiatric care is voluntary. The patients concerned enjoy the same rights as all other people receiving health care. Nonetheless, a system of care without consent makes it possible to provide the necessary care to patients who are not conscious of their mental disorders or of their imperative need for care. This care without patients' consent is regulated by French law, which requires that only facilities participating in public service are licensed to provide it. The law sets conditions to guarantee the protection of the individual's rights and liberties [3].

Three types of care are available in psychiatric healthcare facilities:

- Outpatient management, which designates reception and care performed essentially in medicopsychological centres (CMPs), as well as consultations at other sites, especially those related to liaison psychiatry in health and social establishments;
- Part-time management, more than that provided for outpatients, but without continuous day- and night-time management: centres for part-time therapy (CATTP), therapeutic workshops, day hospitals, and night hospitals;
- Full-time inpatient care in premises enabling care and monitoring 24/7;
- Other types of services can be set up, such as home care, provided essentially as a public service in France.

There are 56,000 beds available for full-time management (but also in family therapy placements, residential aftercare, therapeutic apartments, home hospitalisation, and crisis centres) and 29,000 places for day- or night-hospital care. Outpatient management, mostly in CMPs, is the most frequent form of care [4].

References:

1. LOI n° 2016-41 du 26 janvier 2016 de modernisation de notre système de santé (1) - Légifrance. https://www.legifrance.gouv.fr/jorf/id/JORFTEXT000031912641?r=TWMIky6Doo. Accessed 30 Oct 2020.

2. Ministère de la santé publique et de la population. Circulaire du 15 mars 1960 relative au programme d’organisation et d’équipement des départements en matière de lutte contre les maladies mentales. 1960. http://www.ascodocpsy.org/wp-content/uploads/textes_officiels/Circulaire_15mars1960bis.pdf.

3. LOI n° 2011-803 du 5 juillet 2011 relative aux droits et à la protection des personnes faisant l’objet de soins psychiatriques et aux modalités de leur prise en charge (1) - Légifrance. https://www.legifrance.gouv.fr/jorf/id/JORFTEXT000024312722/. Accessed 30 Oct 2020.

4. Les établissements de santé - édition 2019 - Ministère des Solidarités et de la Santé. https://drees.solidarites-sante.gouv.fr/etudes-et-statistiques/publications/panoramas-de-la-drees/article/les-etablissements-de-sante-edition-2019. Accessed 30 Oct 2020.
